# Topographic correlation between multifocal electroretinography, microperimetry, and spectral-domain optical coherence tomography of the macula in patients with birdshot chorioretinopathy

**DOI:** 10.1186/s12348-019-0188-5

**Published:** 2019-12-28

**Authors:** Rubbia Afridi, Aniruddha Agarwal, Nam V. Nguyen, Muhammad Hassan, Mohammad Ali Sadiq, Quan Dong Nguyen, Yasir J. Sepah

**Affiliations:** 1 Ocular Imaging Research and Reading Center, Menlo Park, CA USA; 2 Byers Eye Institute, Stanford University, Palo Alto, CA USA; 3 Advanced Eye Center, Department of Ophthalmology, Post Graduate Institute of Medical Education and Research (PGIMER), Chandigarh, India

**Keywords:** Birdshot chorioretinopathy, Microperimetry, Optical coherence tomography, Multifocal electroretinogram, Multimodal imaging

## Abstract

**Purpose:**

To correlate the findings of retinal function with multifocal electroretinogram (mfERG), microperimetry (MP), and structural assessments with spectral-domain optical coherence tomography (SD-OCT) in topographically corresponding areas of the macula of patients with birdshot chorioretinopathy (BSCR).

**Methods:**

Patients diagnosed with BSCR by clinical and imaging findings were included in the study. The mfERG was performed using 61 hexagon stimulus patterns grouped into 5 rings (Diagnosys Inc., USA). Individual responses [N1-P1 amplitudes in nanovolt (NV)/degree^2^ and P1 implicit time in milliseconds (msec)] for each hexagon in the central 3 rings (R1, 0°–2.3°; R2,2.3°–7.7°; and R3, 7.7°–12°) were obtained (19 hexagons). MP examination consisted of Polar 3–12° test with 28 points in 3 concentric rings with diameters of approximately 2.3°, 6.6°, and 11.1° from the foveal center. SD-OCT was performed using macular scans of 20° × 20°. The retinal sensitivity values on MP and thickness values of retinal layers were correlated with the responses on the mfERG for each topographically correlated hexagon.

**Results:**

Sixteen eyes of eight patients were included in the study (mean age, 59.87 ± 10.01 years; range, 41–73 years). The amplitudes and the implicit times on mfERG and retinal sensitivities on MP were decreased for each of the 19 hexagons. Considering retinotopically matched points, there was correlation between the retinal sensitivities and mfERG implicit times and response amplitudes in all three rings. The thickness of the retinal pigment epithelium showed modest correlation with the mfERG parameters (*ρ* = 0.29; *p* = 0.04). The structural changes on SD-OCT, such as IS-OS disruption, were associated with changes in the mfERG trace arrays.

**Conclusions:**

The structural and functional assessments in retinae of eyes with BSCR suggest that each imaging tool may be capturing unique aspects of retinal dysfunction. Multimodal imaging may allow detailed analyses of retinal damage at various corresponding loci. These findings are important when considering the use of these techniques in BSCR.

## Background

Since its first description in 1980 [1], birdshot chorioretinopathy (BSCR) remains a management challenge due to its chronic progressive nature resulting in significant chorioretinal damage. Often, the disease course may appear to be clinically stable as the Snellen’s visual acuity may not worsen. This led clinicians to believe that BSCR represents an inflammatory uveitic entity with a self-limiting, benign course [2, 3]. However, by the time central visual acuity is affected, irreversible atrophy and degeneration may have already occurred [4].

In the past decade, significant advances have been made in diagnostic techniques that allow objective measurement of the chorioretinal structural and functional alterations. Serial assessment of patients with BSCR using electroretinograms [5, 6] and visual field testing [7] has demonstrated a chronic, relentless course of the disease even in the absence of clinically detectable intraocular inflammation [8]. In addition, studies using noninvasive imaging techniques such as spectral-domain optical coherence tomography (SD-OCT) have shown significant atrophy of the retinal layers of the macula and mid-periphery among patients with BSCR [9, 10]. Functional loss due to retinal cell death may manifest as early functional change, which may be detected using microperimetry [11], a technique that allows quantification of macular sensitivity. Thus, the preferred long-term follow-up approach of patients with BSCR consists of multimodal imaging with an aim to detect and treat early signs of pathological damage [12].

Techniques such as electroretinography, microperimetry, and optical coherence tomography are available to determine the extent and severity of structural and functional chorioretinal damage in eyes with BSCR, and there is scarcity of literature that has assessed the relationship between them. Detailed retinal topographic correlation may allow comprehensive assessment of the role of each of the abovementioned technology. In addition, multimodal imaging correlation may enable identification of biomarkers that may serve to monitor the disease progression and response to treatment.

The index study was conducted to study correlation between implicit times and amplitudes of mfERG, retinal point sensitivity using microperimetry, and thickness of retina of spectral-domain OCT at the same retinal location.

## Material and methods

For the purpose of the index study, images from patients of BSCR undergoing diagnostic testing and treatment at the Retina and Uveitis Clinic between January 2013 and January 2015 were analyzed. Institutional Review Board (IRB) clearance was obtained, and the study adhered to the tenets of the Declaration of Helsinki. All the study procedures were compliant as per the Health Insurance Portability and Accountability Act (HIPAA) of 1996.

The inclusion criteria for the study subjects included a diagnosis of BSCR ascertained by clinical findings, slit-lamp biomicroscopy, and indirect ophthalmoscopic visualization of characteristic yellow-orange ovoid chorioretinal lesions with mild vitritis, as per the International Consensus Conference on BSCR [13] by an uveitis specialist (Q.D.N). Only those patients undergoing diagnostic testing with all the three modalities—mfERG, MP, and SD-OCT—were included in the study. The exclusion criteria for the study subjects were best-corrected visual acuity (BCVA) < 20/100, poor central fixation (fixation within 2° ≤ 75%), and mean spherical refractive error of ≥ 3 diopters. The other exclusion criteria were presence of choroidal neovascular membrane, significant optic nerve disease or glaucoma, patients unable to cooperate for the test and provide reliable results, and patients with any neurological or systemic disease that can affect vision and/or the test results. Patients on systemic treatment with agents that can affect macular function, such as hydroxychloroquine, or those participating in clinical trials for investigational new drugs in uveitis were excluded from the study.

BCVA assessment was performed by independent masked observers. mfERG was obtained approximately 2 weeks prior to the scheduled clinic visit. BCVA assessment and MP were obtained prior to any other imaging on the day of the clinic visit to avoid bleaching of the photoreceptors. SD-OCT was performed at the clinic visit. In this study, only those patients who had undergone prior testing with MP and had provided reliable results were included in the study (*vide infra*).

### Multifocal electroretinogram

mfERG was obtained based on the guidelines established by the International Society of Clinical Electrophysiology of Vision (ISCEV) [14]. Electrophysiology testing was performed using the Espion V6, Diagnosys LLC (Diagnosys Inc., USA) device by a single trained operator masked to the clinical and other imaging data. Pupillary dilation was performed using 1% tropicamide and 2.5% phenylephrine to obtain a pupillary diameter of at least 6 mm. The patient was seated in a room with moderate or dim room lights for at least 20 minutes prior to the procedure. The mfERG was performed using the disposable Dawson-Trick-Litzkow (DTL) electrodes placed at the inferior limbus to avoid loss of amplitude. Sixty-one hexagon stimulus pattern mfERG was performed with a frame frequency of 75 Hz. The luminance in the light stage was 1000 cd/m^2^. The central fixation target of a cross was used. The mfERGs were obtained with a band pass filter of 10–100 Hz and a base period of 13.3 msec. First-order kernel responses were reported, and the spatial averaging, noise rejection, and smoothing were switched off since they can obscure small, localized changes. mfERG results consisting of trace arrays with N1-P1 amplitudes and P1 implicit times for each individual hexagon were obtained.

**Fig. 1:**
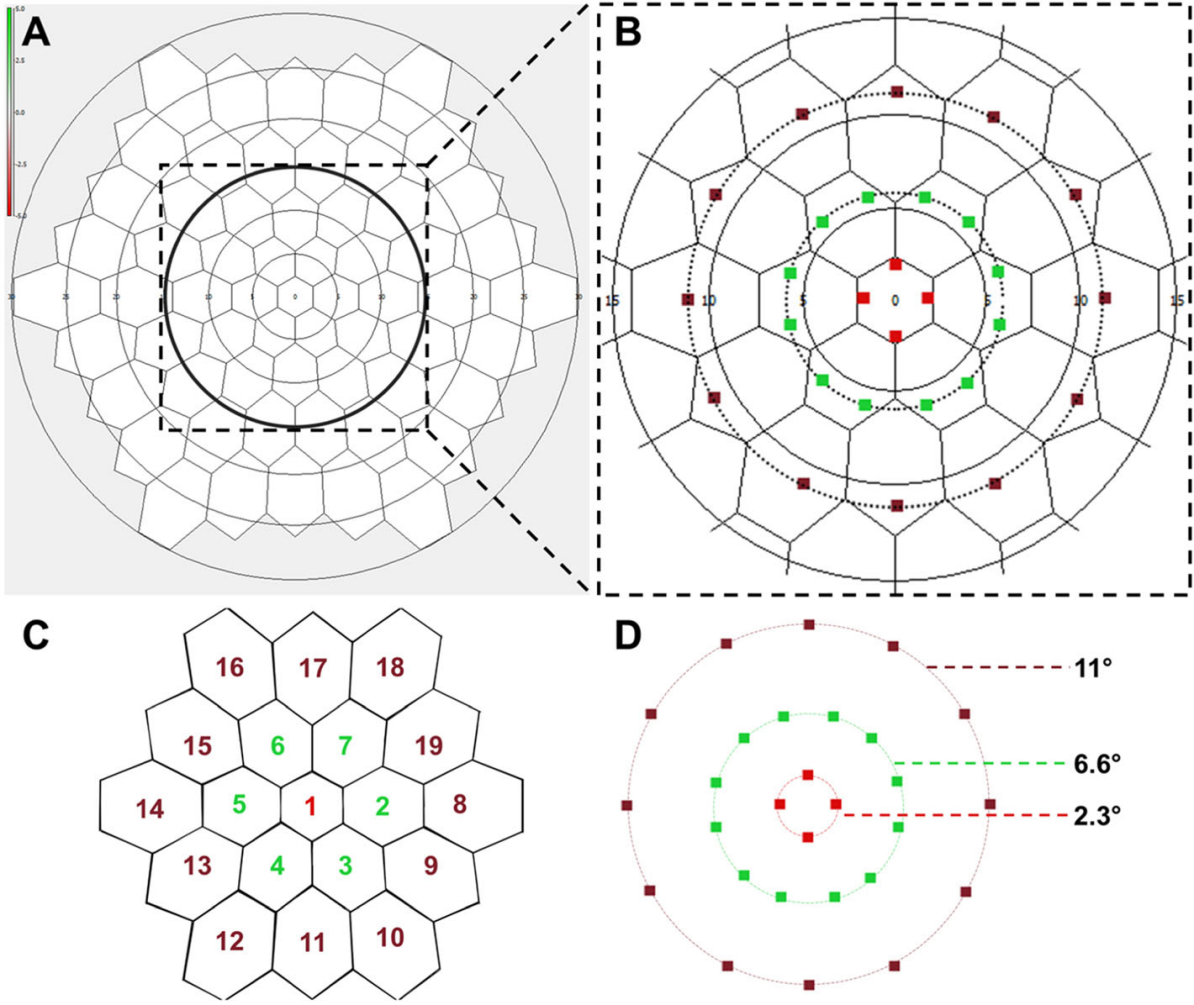
An overlay and numbering scheme of the multifocal electroretinography (mfERG) hexagons and the microperimetry (MP) stimuli points. The hexagons (*n* = 19) selected for the analysis are marked by a circle in **a**. In **b**, these 19 selected hexagons are overlapped with the MP test points using the exact distances from the foveal center. Thus, four central test points on MP lie on the inner hexagon (red color), two test points lie inside each hexagon in the second ring (green color), and one MP test point lies inside each hexagon in the outer ring (brown color). The nomenclature of the hexagons is depicted in **c**, where the central hexagon is labeled as 1. The distances from the foveal center of the MP test points is shown in **d**

The stimuli of mfERG are usually grouped into five concentric rings, and the summed responses divided by the total number of hexagons provide an averaged response/hexagon. In the present study, individual responses for each hexagon in the central 3 rings were obtained (19 hexagons). The central ring (R1) extends from approximately 0° to 2.3° from the foveal center, the second ring (R2) measures responses from 2.3° to 7.7° of the foveal center, and the third ring (R3) measures responses from 7.7° to 12° from the foveal center (Fig. 1).

### Microperimetry

MP examination was performed using Optos SLO microperimetry (Optos Inc., USA). Adequate pupillary dilation was ensured prior to the test. All the patients were explained the test technique prior to the testing. Prior to the perimetry exam, fixation test was performed for each eye (monocular testing) for 20 seconds. In this study, Polar 3–12° test was used which consists of 28 test points and 4-2 testing strategy. The stimuli are arranged in 3 concentric rings consisting of central 4 test points arranged in a ring measuring approximately 2.3° in diameter around the foveal center, 12 points in a ring of 6.6° diameter, and 12 points in a ring 11.1° diameter. Goldmann size III stimuli were presented for 200 msec. During the test, the device assesses fixation by a retinal tracker using a scanning laser ophthalmoscope with a super-luminescent diode laser of 850 nm wavelength. Tests with false positive rates ≥ 20% and a false negative rate of ≥ 20% were regarded as unreliable.

### Spectral-domain optical coherence tomography

SD-OCT scans were performed for the study subjects using Heidelberg Spectralis HRA + OCT (Heidelberg Engineering, Heidelberg, Germany). Macular scans of 20° × 20° were obtained for all the patients during the clinic visit by independent masked operators. The scan consists of 25 horizontal B-scans encompassing the macula with a distance of approximately 245 μm per line scan. A minimum of 25 averaged ART scan protocol was followed for obtaining the cross-sectional images of the macula. In addition, 20 eyes of 20 age-matched control subjects with no known ocular disease were included in the study, and their macular OCT scans were analyzed. OCT parameters of these healthy subjects were compared with those of patients with BSCR.

### Correlation between mfERG and MP

The responses from each hexagon [NI-PI amplitudes in nanovolt (NV)/degree [2] and P1 implicit time in milliseconds (msec)] were obtained. The responses from the 3 inner rings (19 hexagons) were correlated to the MP as shown in Fig. 1. The central four test points on the MP were averaged and represented the central hexagon on the mfERG. Two responses from the middle ring on the MP were averaged to represent a hexagon belonging to R2 on mfERG, where a single test point in the outermost ring on MP corresponded to the R3 on mfERG. The overlap scheme along with the nomenclature of the hexagons on the mfERG (numbers 1–19) is depicted in Fig. 1 [15]. The retinal sensitivity values on MP were correlated with the amplitudes and implicit times on the mfERG for each topographically correlated hexagon.

### Correlation of SD-OCT with mfERG and MP

**Fig. 2:**
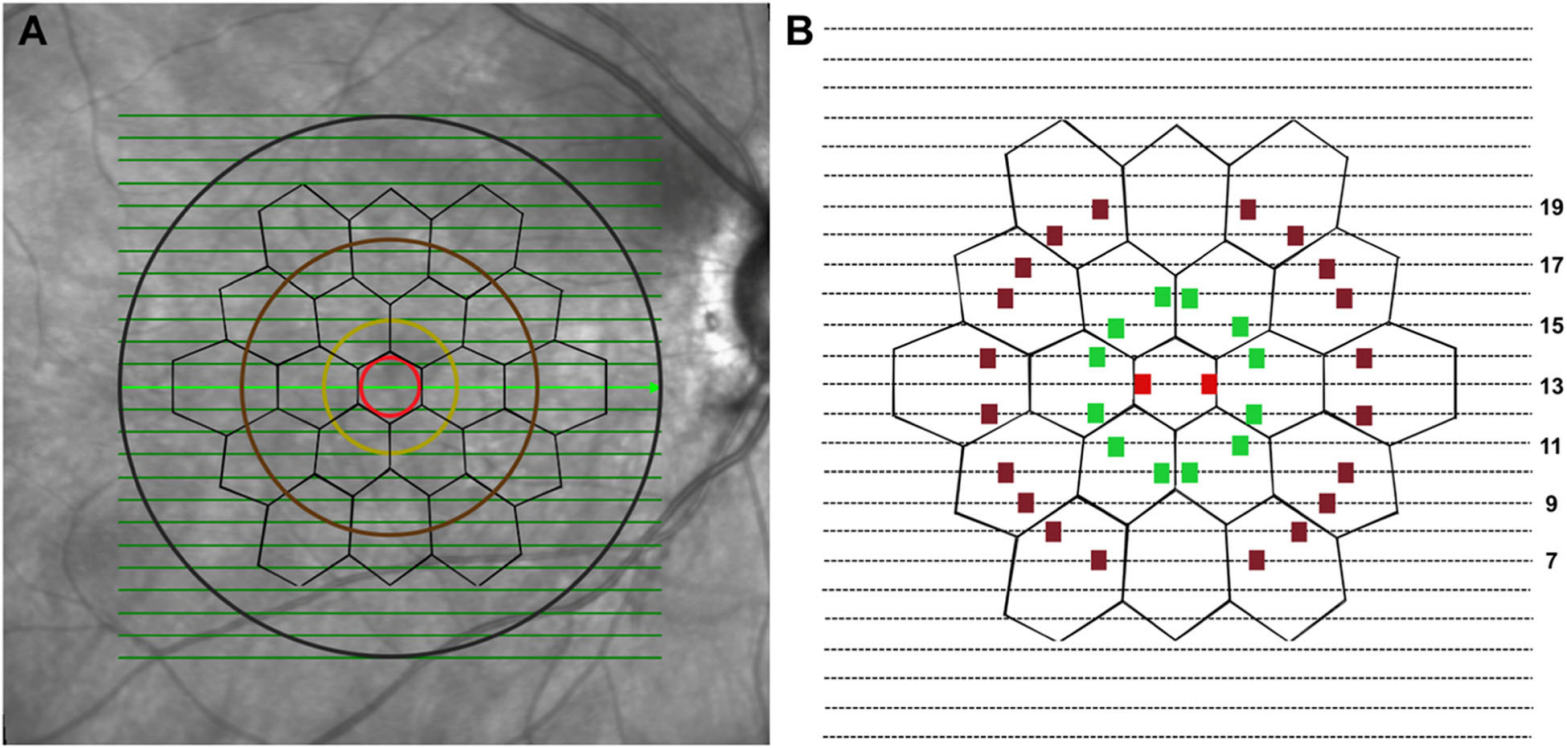
The correlation of spectral-domain optical coherence tomography (SD-OCT), multifocal electroretinography (mfERG) hexagons, and the microperimetry (MP). **a** The SD-OCT line scans intersect various hexagons of the mfERG as shown. Concentric circles with diameters of 2.3°, 6.6°, and 11.1° (representing the topographic locations of the MP test points) are also shown in red, green, and brown circles. Analysis of the retinal layers was performed at the locations where the superimposed circles intersected the line scans of the SD-OCT. Two points were selected, and their values were averaged within 17 hexagons (hexagon numbers 11 and 17 were excluded since the line scans did not intersect the circles representing the MP test points) so that a total of 34 areas were analyzed for each SD-OCT scan (**b**)

The images of the SD-OCT were exported in a tagged image file format (TIFF). Using Adobe Photoshop CS6 (Adobe Creative Suite, Adobe Inc., USA), and the mfERG hexagons of the central three rings (R1–R3) were superimposed on the infrared image of the SD-OCT. Concentric circles with diameters of 2.3°, 6.6°, and 11.1° (representing the topographic locations of the MP test points) were drawn (Fig. 2a). Analysis of the retinal layers was performed at the locations where the superimposed circles intersected the line scans of the SD-OCT. Two points were selected, and their values were averaged within 17 hexagons (hexagon numbers 11 and 17 were excluded since the line scans did not intersect the circles representing the MP test points) so that a total of 34 areas were analyzed for each SD-OCT scan (Fig. 2b). At these topographically correlated locations, thicknesses of the retinal pigment epithelium (RPE)-Bruch’s membrane complex and full retinal thickness (FRT) were measured using the linear caliper tool on the Heyex Eye Explorer v5.6 (Heidelberg Engineering, Heidelberg, Germany). The TIFF images were used to assist the graders in localizing the area selected for analysis on the Heyex Eye Explorer. The SD-OCT measurements were performed by two independent graders (A.A and R.A) and compared for inter-observer agreement.

In addition to quantitative analysis, qualitative analysis on the SD-OCT was also performed. This consisted of identification of loss of the integrity of the photoreceptor inner segment-outer segment (IS-OS) junctions, RPE layer, presence of chorioretinal lesions, and other inner retinal pathologies. In cases with large chorioretinal lesions resulting in distortion and poor identification of retinal layers, SD-OCT analysis was avoided.

### Statistical analysis

Statistical analysis was performed using SAS data package. The correlation between the amplitudes and implicit times on mfERG with the retinal sensitivity values on MP and with the retinal thicknesses on SD-OCT was tested for each of the topographically corresponding hexagons using Pearson’s correlation coefficient. Similarly, the retinal sensitivity values on MP were correlated with the retinal layer thickness values on SD-OCT. BCVA was correlated with the central hexagon on mfERG, average of the central four points on MP, and the retinal layer thicknesses in the corresponding topographic area. A *p* value of < 0.05 was considered to be statistically significant.

## Results

Sixteen eyes of eight patients (one male) met our inclusion criteria, and their clinical data and images were analyzed. The mean age of all the subjects was 59.87 ± 10.01 years. All the patients diagnosed were Caucasian. All the patients included in the study were HLA-A29 positive. The mean duration of the disease was 24.12 ± 6.4 months. The management of the patients included oral corticosteroids (all the patients were on maintenance dose of ≤ 10 mg/day oral prednisone at the time of enrollment) and systemic immunosuppressive agents (mycophenolate mofetil or azathioprine). The mean visual acuity at the time of the imaging evaluation was 0.96 ± 0.62 LogMAR units. All the participants included in the study had reliable MP imaging and adequate quality SD-OCT and mfERG scans. In order to determine the normal values of the SD-OCT retinal layer thicknesses, 20 eyes of 20 healthy volunteers (59.0 ± 7 years) were included in the study.

### Comparison of mfERG and microperimetry

The mean mfERG values in the three rings (R1, R2, and R3) are represented in Table 1. Compared to the normative database (collected by the mfERG laboratory by the authors), the values of implicit times were increased, and amplitudes were decreased compared to the normative database in all three rings concentric from the foveal center. Considering the retinotopically matched points, there was a trend towards negative correlation with the MP sensitivity values in R1 and R2 but did not reach statistical significance (R1, rho = − 0.46; *p* = 0.07; R2, rho = − 0.14; *p* = 0.11) and did not show any relationship at R3 (rho = 0.19; *p* = 0.48). The mfERG amplitudes showed moderate positive correlation with MP sensitivities in the inner two rings but did not show statistical significance in the outer third ring (R1, rho = 0.63; *p* = 0.008; R2, rho = 0.64; *p* = 0.007; R3, rho = 0.46; *p* = 0.07).

**Table 1:** Mean values of the multifocal electroretinography, microperimetry, and retinal layer thicknesses obtained using spectral-domain optical coherence tomography among subjects with birdshot chorioretinopathy included in the study

	Inner ring (R1)	Middle ring (R2)	Outer ring (R3)
Electroretinography parameters			
Implicit time (ms)	35.9 ± 9.0	35.6 ± 4.8	35.3 ± 2.8
Amplitude (nV/deg^2^)	23.0 ± 11.6	13.0 ± 3.2	9.4 ± 3.5
Microperimetry			
Macular sensitivity (dB)	9.9 ± 5.3	11.2 ± 5.1	10.6 ± 3.7
Spectral-domain optical coherence tomography			
Full retinal thickness (μm)	250.4 ± 21	253.4 ± 22	231.0 ± 22
RPE-Bruch’s layer Thickness (μm)	41.0 ± 5.1	33.7 ± 4.3	24.1 ± 3.9

*The rings R1, R2, and R3 represent 0°–2.3° from the foveal center, 2.3°–7.7° from the foveal center, and 7.7°–12° from the foveal center, respectively

### Comparison of SD-OCT with microperimetry and mfERG

The FRT and the thickness of the RPE layer among subjects were compared to normal healthy controls (mean age, 58.6 ± 6.86 years; *p* = 0.4 comparing mean age with BSCR subjects). The mean FRT among subjects with BSCR is provided in Table 1. The mean FRTs among healthy controls were significantly higher than those of BSCR patients at 308 ± 70 μm (*p* < 0.05) (R1), 296 ± 67 μm (*p* < 0.05) (R2), and 299 ± 71 μm (*p* < 0.01) (R3), respectively. The mean RPE thickness was also lower among subjects with BSCR compared to healthy controls (20.87 ± 8 μm versus 36.0 ± 10 μm at R1 (*p* = 0.01); 20.87 ± 8 μm versus 34.1 ± 10 μm at R2 (*p* < 0.05); and 17.81 ± 6 μm versus 34.0 ± 9 μm at R3 (*p* < 0.01)).

The mean values of FRT and RPE thicknesses were correlated with the retinotopically correlated values of retinal sensitivities on MP at R1, R2, and R3. The correlation between MP values and FRT thickness did not show any significant correlation (R1, rho = 0.06; *p* = 0.82; R2, rho = − 0.25; *p* = 0.35; R3, rho = 0.14; *p* = 0.14). The correlation between MP and RPE thickness also did not show any significant correlation at the three hexagonal rings (R1, rho = − 0.27; *p* = 0.31; R2, rho = − 0.16; *p* = 0.55; R3, rho = − 0.03; *p* = 0.91).

Table 2 depicts the correlation between MP, ERG, and SD-OCT values.

**Table 2:** Correlation between the mean retinal sensitivity on microperimetry with the parameters on multifocal electroretinography and retinal layer thicknesses obtained using spectral-domain optical coherence tomography among subjects with birdshot chorioretinopathy included in the study

	Inner ring (R1)	Middle ring (R2)	Outer ring (R3)
Correlation of bicroperimetry with electroretinography			
MP with implicit time	− 0.46 (0.07)	− 0.14 (0.11)	0.19 (0.48)
MP with amplitude	0.63 (0.008)	0.64 (0.007)	0.46 (0.07)
Correlation of microperimetry with spectral-domain optical coherence tomography			
MP with FRT	0.06 (0.82)	− 0.25 (0.35)	0.14 (0.14)
MP with RPE	− 0.27 (0.31)	− 0.16 (0.55)	− 0.03 (0.91)

*The rings R1, R2, and R3 represent 0°–2.3° from the foveal center, 2.3°–7.7° from the foveal center, and 7.7°–12° from the foveal center, respectively

*FRT* full retinal thickness; *MP* Microperimetry; *RPE* retinal pigment epithelium-Bruch’s membrane complex

The values are indicated in rho (*p* value)

**Fig. 3:**
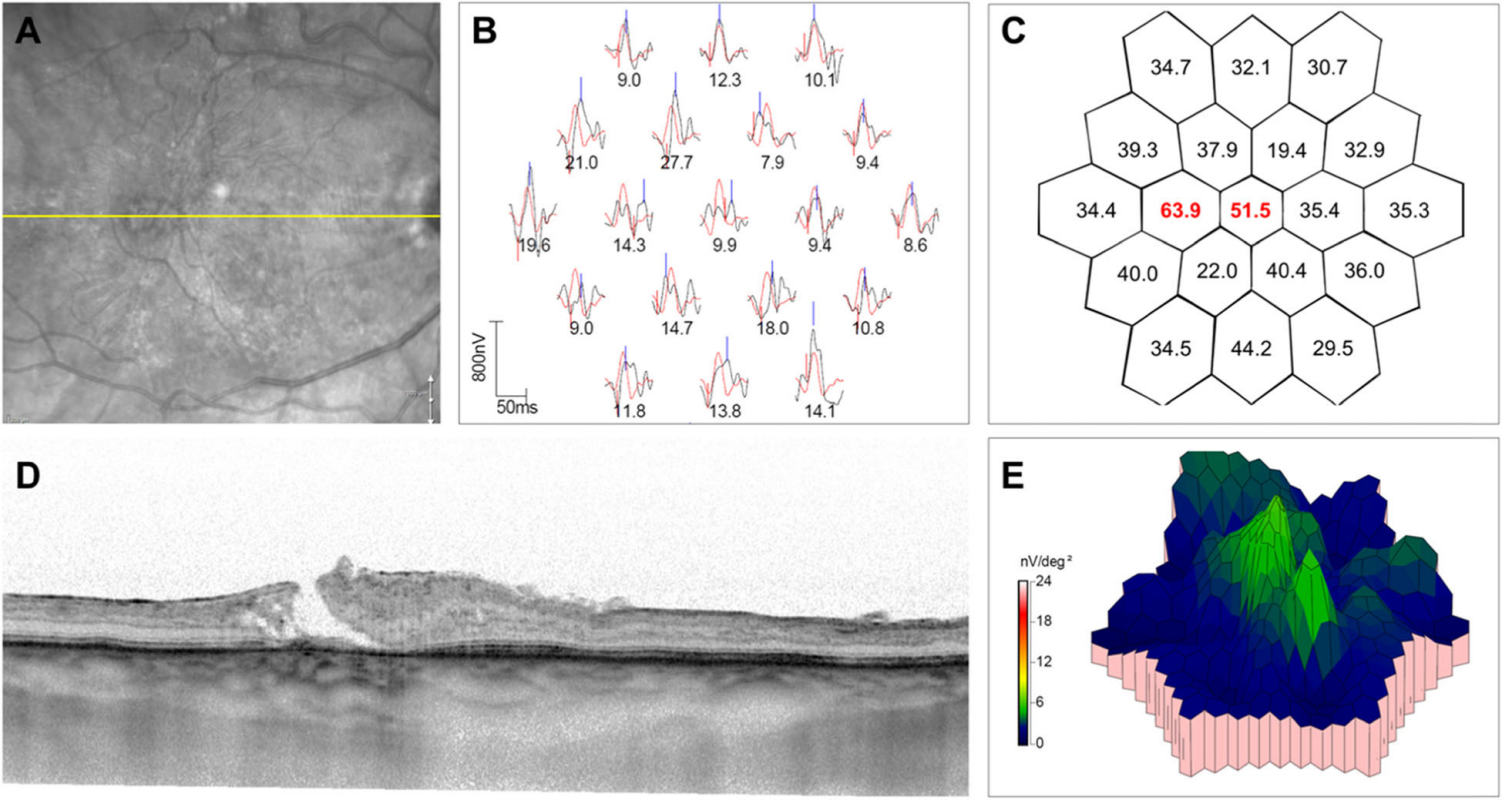
Correlation between the spectral-domain optical coherence tomography (SD-OCT) line scans and multifocal electroretinography (mfERG) of a patient with birdshot chorioretinopathy (**a–d**). The area through which the line scan passes is shown in the infrared image in **a**. The OCT line scan (**d**) shows disruption of the inner and outer retinal layers at an area temporal to the foveal center. The mfERG trace arrays show abnormal waveforms at the retinotopically correlated points represented by the area of tissue disruption on OCT (**b**). In addition, the numeric values below the abnormal waveforms show reduced amplitude density at these areas of interest. The hexagons in (**c**) (2D topography maps) depict the implicit times at each of the tested areas. In the area of SD-OCT retinal layer disruption, there are increased implicit times noted in red. The color map also shows blunted mfERG response (**e**)

In comparison with SD-OCT with mfERG, the two tests showed decreased structure/function for all the patients. A case example of correlation between SD-OCT changes and mfERG implicit time and amplitude changes is provided in Fig. 3. The figure represents a patient diagnosed with BSCR on immunosuppression for the past 2 years. The SD-OCT scan shows an area of inner and outer retinal tissue loss, which correlates with abnormal mfERG waveforms, increased mfERG implicit time, and decreased amplitudes. This example illustrates that the loss detected on mfERG correlates well with changes on SD-OCT.

### Correlation of best-corrected visual acuity

The mean BCVA for all the patients was 0.96 ± 0.62 LogMAR units. There was a strong positive correlation between the BCVA and mfERG implicit times (in R1) with Pearson’s correlation coefficient (rho = 0.91; *p* < 0.001), which meant that lower BCVA values were associated with higher mfERG implicit times. However, there was no correlation between the mean BCVA values and the mfERG amplitudes at R1 (rho = 0.06; *p* = 0.83). Mean BCVA was negatively correlated to macular sensitivity on MP at R1 (rho = − 0.31; *p* = 0.24) suggesting that lower BCVA was associated with poorer MP sensitivity values though it did not reach statistical significance. Correlation between BCVA and FRT at R1 was also negatively correlated (rho = − 0.73; *p* = 0.001) (thinner retinae were associated with lower BCVA). The thickness of the RPE layer at R1 was negatively correlated with BCVA (rho = − 0.37; *p* = 0.15) (thinner RPE was associated with lower BCVA) (but it did not reach statistical significance).

Table 3 depicts the correlation of BCVA with MP, ERG, and SD-OCT values.

**Table 3:** Correlation between best-corrected visual acuity with the parameters on multifocal electroretinography, microperimetry, and retinal layer thicknesses obtained using spectral-domain optical coherence tomography among subjects with birdshot chorioretinopathy included in the study (only in the central ring)

	Inner ring (R1)
Correlation of BCVA with bicroperimetry	
BCVA with MP	− 0.31 (0.24)
Correlation of BCVA with electroretinography	
BCVA with implicit time	0.91 (<0.001)
BCVA with amplitude	0.06 (0.83)
Correlation of BCVA with spectral-domain optical coherence tomography	
BCVA with FRT	− 0.73 (0.001)
BCVA with RPE	− 0.37 (0.15)

*The ring R1 represents 0°–2.3° from the foveal center

*BCVA* best-corrected visual acuity; *FRT* full retinal thickness; *MP* microperimetry; *RPE* retinal pigment epithelium-Bruch’s membrane complex

The values are indicated in rho (*p* value)

## Discussion

BSCR is a rare cause of posterior uveitis predominantly observed in middle-aged Caucasian females resulting in distinct choroidal lesions, vasculitis, and mild vitreous inflammation. Ethnic predominance correlates probable disease association with HLA-A29 subtypes. Central vision loss occurs later in the disease process and is accompanied by severe irreversible structural changes. It is therefore important to identify biomarkers of structural and functional decline in eyes with BSCR that may allow for better understanding of the underlying pathological processes and may serve as surrogate markers of disease progression in the presence of good central vision.

Mean FRT and RPE thickness in the study population were significantly lower than the corresponding mean values in healthy controls. RPE and overall retinal thinning were observed. Decreased FRT and RPE values thus confirm structural changes associated with the disease process. Mean values of FRT and RPE thickness, when compared with the retinal sensitivities at the MP test points, showed no significant correlation. This correlation between layer thickness and retinal sensitivity indicates that retinal thickness did not affect the sensitivity at/around the lesion area – contrary to the results reported previously in uveitis subjects [16]. It is possible that MP is not sensitive enough to detect changes associated with such small structural changes on OCT. The mean mfERG values from the three rings were compared with corresponding mean MP values from the three rings using Pearson’s correlation. A moderate correlation was reported among mean mfERG amplitudes and MP sensitivities. Thus, structural damage that occurs in BSCR results in loss of retinal function in the retinotopically corresponding points. These findings highlight the relevance of mfERG in BSCR to demonstrate retinal functional damage, which correlates with findings on MP.

The mean BCVA was calculated for the study population in R1 which corresponds to the central vision. Increased implicit times on mfERG corresponded to poor BCVA scores. Low MP sensitivity values were observed to have a negative correlation with BCVA, i.e., low MP sensitivity/low BCVA. Similar correlation between retinal sensitivity and BCVA was observed previously in uveitis subjects [16]. OCT values of a decreased FRT and RPE thickness also correspond to low BCVA. Thus, this indicates that central visual function (i.e., BCVA) affected during disease process correlates to structural damage on mfERG and OCT.

Each imaging techniques used in the study provide unique functional or structural information related to BSCR, true to each modality. While OCT imaging provides a picture of disease-related structural loss, BCVA provides the functional compromise due to the structural loss. MP and mfERG also demonstrate loss of photoreceptor sensitivities and compromised retinal cellular electrophysiological functions. This index study is the first to our knowledge to compare the characteristics of multimodal imaging in clinical use for BSCR. The study outlines the possible relationship between BSCR lesion as seen on OCT, the effects of the disease on the electrophysiology of area encompassed by the lesion, and the compromise in the retinal function at the lesion site as assessed by microperimetry. From the results of the study, a direct correlation between different imaging techniques was observed. For most part, the correlation between structure loss and functional compromise follows the norms of proven concept of the simultaneous structural and functional loss, e.g., central vision loss as seen from the BCVA values in R1, which was seen to correlate directly with structural loss and retinal sensitivity loss in R1.

Considering the interesting outcomes, there is need for further evaluation of disease progression on the structural, functional, and clinical outcomes on a larger study population. Human error cannot be excluded in this study as observations and evaluations were performed manually. A more reliable outcome of multimodal analysis can be ensured by making the processes automated (automated superimposition of MP and ERG on *en-face* OCT, OCT layers thickness calculation).

## Conclusions

Multimodal imaging is well-known to the diagnosis and management of uveitis. Correlation among characteristic findings of multimodal imaging in BSCR directly point towards structural and functional relationship of the disease process. Findings from SD-OCT, mfERG, and MP show a positive correlation among themselves and with visual acuity gain or loss, as a predictor of functional outcome clinically.

## Data Availability

The datasets used and/or analyzed during the current study are available from the corresponding author on reasonable request.

## Abbreviations

BCVA: Best-corrected visual acuity
BSCR: Birdshot chorioretinopathy
DTL: Dawson-Trick-Litzkow
HIPPA: Health Insurance Portability and Accountability Act
ISCEV: International Society of Clinical Electrophysiology of Vision
mfERG: Multifocal electroretinogram
MP: Microperimetry
SD-OCT: Spectral-domain optical coherence tomography
TIFF: Tagged image file format
